# Temporal dynamics of inflammatory proteins in the pre-symptomatic phase of rheumatoid arthritis related to seroconversion

**DOI:** 10.1186/s13075-026-03917-4

**Published:** 2026-10-06

**Authors:** Anton Ahlsved, Anders Lundquist, Kristina Lejon, Solbritt Rantapää-Dahlqvist, Mikael Brink

**Affiliations:** 1 Department of Public Health and Clinical Medicine, Rheumatology Umeå University, Umeå, SE-901 85 Sweden; 2 Department of Statistics, USBE, Umeå University, Umeå, Sweden; 3 Department of Clinical Microbiology, Umeå University, Umeå, Sweden

## Abstract

**Objective:**

Autoimmune processes in rheumatoid arthritis (RA) begin years before clinical symptom onset. This study aimed to analyse inflammation-related proteins in individuals during the pre-symptomatic phase, in relation to healthy controls and patients with early RA, and their relationship to seroconversion.

**Methods:**

Plasma samples collected prior to symptom onset from individuals who later developed RA (*n* = 419), healthy population controls (*n* = 49), and patients with early RA at diagnosis (*n* = 68) were obtained from a biobank. All samples were analysed for 92 inflammation-related proteins using a proximity extension assay and for anti-CCP2 antibodies and rheumatoid factor. After quality control 77 proteins remained. To identify differentially expressed proteins and enriched pathways, statistical analyses including logistic regression, generalized additive models, and network enrichment analysis was applied.

**Results:**

Four proteins (MMP10, IL6, CCL19, and IL10RB) showed a higher concentration in pre-symptomatic individuals compared to controls (*p* < 0.05). Twelve proteins were associated with seropositivity in the pre-symptomatic phase and network enrichment analysis of these revealed associated pathways related to the complement system and cytokine activity. Three proteins (CXCL10, CCL7, and CDCP1) were consistently associated with seroconversion and showed increasing trends approaching symptom onset in individuals who seroconverted, but not in individuals who remained seronegative. Proteins associated with early seroconversion were associated with T-cell activity, whereas many of the proteins associated with late seroconversion were associated with bone remodelling, mineral metabolism, and collagen degradation.

**Conclusion:**

Proteomic alterations are detectable years before RA onset and linked to autoantibody development. Seroconverters differ from those who remain seronegative even in the pre-symptomatic period, supporting the notion that seropositive and seronegative RA represent biologically distinct entities from the earliest stages of the pathogenesis.

**Supplementary Information:**

The online version contains supplementary material available at https://doi.org/10.1186/s13075-026-03917-4.

## Introduction

Rheumatoid arthritis (RA) is a chronic progressive autoimmune disease that predominately affects the joints, and is characterized by symmetrically swollen and tender joints, morning stiffness, tenosynovitis and destruction of the affected joints [1]. RA is the most common inflammatory joint disease and has a lifetime prevalence of around 0.5% [2]. The pathophysiology of the disease is not fully understood, where two thirds of all RA patients are positive for rheumatoid factor (RF) and/or antibodies against citrullinated protein (ACPA) at diagnosis [3].

The autoimmune processes begin years before clinically apparent arthritis is evident and previous studies have shown that autoantibodies associated with RA; ACPA and RF are present years before symptom onset [4, 5]. As shown by our group earlier, one third of all pre-symptomatic individuals are positive 5 years before symptom onset [6]. Furthermore, levels of cytokines and chemokines differ between pre-symptomatic individuals and controls, in a time to symptom onset dependent manner [7–9]. In addition, it has been demonstrated that the levels of ACPA and the number of specific reactivities increase over time in the pre-symptomatic period, i.e. epitope spreading [6, 10, 11]. On the contrary, no significant increase has been observed for ACPA concentrations or cytokine gene expression levels in the time period between symptom onset and diagnosis [12]. This supports the notion that autoimmunity is established prior to symptom onset, reinforcing the importance of studying the pre-symptomatic phase to better understand the pathophysiological mechanisms driving RA.

While the precise mechanism behind autoimmune processes leading to seropositivity remains unknown, researchers have pinpointed genetic as well as environmental factors e.g. smoking as significant contributors [13]. Thus, the risk of future seropositive RA is increased if smoking is combined with genetic risk factors (HLA-DRB1 shared epitope) through a gene-environment interaction [14, 15].

In the present study, our aim was to analyse inflammation-related proteins in individuals before symptom onset, in relation to early RA patients and population controls. Moreover, we wanted to explore the association between seroconversion and these proteins in a nested case-control cohort with samples from the pre-symptomatic period using a multiplex protein assay.

## Material & methods

### Subjects

A case-control study was performed using plasma samples from the cohorts of the medical biobank of Northern Sweden. The inclusion criteria, storage conditions, and contents of the biobanks have been described earlier [16]. The register of early RA patients at the Department of Rheumatology, Umeå University Hospital, comprising patients fulfilling the American college of Rheumatology 1987 diagnostic criteria for RA [17] and with information regarding self-reported onset of symptom date, were co-analysed with the registers of the Medical Biobank of northern Sweden. The early RA patients were carefully asked for the time point of onset of the actual musculoskeletal symptoms or if these symptoms had appeared earlier in life. For patients who gave a vague answer on the time point it was antedated by 3 months or if the time point was uncertain the patient was excluded. For all early RA patients the medical records were carefully evaluated and information about the presence of anti-CCP and RF was collected.

A total of 493 samples from pre‑symptomatic individuals were initially assessed, of which 474 samples from 419 individuals were retained for analysis, including 55 individuals sampled twice during the pre‑symptomatic period. Exclusions were applied using a predefined priority order (quality control failure, dried source‑plate sample, insufficient volume, PCA outlier), resulting in exclusion of seven samples due to quality control failure, six due to dried source‑plate samples, five due to insufficient sample volume, and one individual due to atypical clustering in unsupervised principal component analysis of Olink data. 50 control samples were identified from the Medical Biobank, of which one was excluded due to a dried source‑plate sample, leaving 49 control samples. Among RA patients, 73 samples were initially available; one was excluded due to lack of a corresponding pre‑symptomatic sample, and among the remainder three were excluded due to quality control failure and one due to a dried source‑plate sample, resulting in 68 RA patients with one sample each included in the final analyses.

The median time from sample collection to symptom onset was 4.35 years in the pre-symptomatic group. There were no statistically significant differences in sex between the controls, the pre-symptomatic samples, and patients. Those who had two samples during the pre-symptomatic period were mainly women (94.5%) compared to 68% in those with only one sample. No significant difference in age at diagnosis, antibody status, smoking status or HLA shared epitope status could be shown between those who had two sample versus one sample (Table 1).

**Table 1:** Characteristics of pre-symptomatic individuals, early RA patients, controls, and pre-symptomatic cases with one or two samples

	Case				Individuals contributing with one or two pre-symptomatic samples		
	Controls	Pre-symptomatic individuals	Early RA Patients	*P*-value^1^	One sample	Two samples	*P*-value^2^
n	49	419	68		364	55	
Female n (%)	35 (71.4)	299 (71.5)	44 (64.7)	0.5	247 (68.0)	52 (94.5)	< 0.001
Time to symptom onset (median [IQR])	-	-4.4 [-6.8, -2.4]	-	-	-4.7 [-7.0 -2.5]	-2.8 [-4.3, -1.6]	< 0.001
Age at sampling (median [IQR])	56.7 [50.0, 60.0]	50.5 [49.7, 60.0]	59.1 [51.4, 63.4]	< 0.001	50.3 [49.4, 60.0]	59.7 [50.7, 60.4]	0.004
Age at symptom onset (median [IQR])	-	57.9 [51.1, 63.6]	58.7 [51.0, 63.0]	0.5	56.9 [50.8, 63.4]	61.8 [53.8, 65.2]	0.04
Rheumatoid Factor positive, n (%)	1/27 (3.7)	152/416 (36.6)	56/68 (82.4)	< 0.001	130/361 (36.1)	22 /55(40.0)	0.68
Anti-CCP2 positive, n (%)	2/49 (4.1)	176/418 (42.2)	51/68 (75.0)	< 0.001	148/363 (40.9)	28/55 (50.9)	0.2
Ever-smoker, n (%)	17/48 (35.4)	268/418 (64.0)	49/68 (72.1)	< 0.001	233/363 (64.4)	34/55 (61.8)	0.8
HLA-shared epitope, n (%)	-	271/410 (66.3)	43/68 (63.2)	-	233/355 (65.5)	39/55 (70.9)	0.5

^1^Calculated using the chi-squared test for categorical variables and the Kruskal-Wallis test for continuous variables, across all three groups

^2^Calculated using the chi-squared test for categorical variables and Mann-Whitney U test for continuous variables, between the individuals with one respectively two samples

### Seroconversion subgroups

**Fig. 1:**
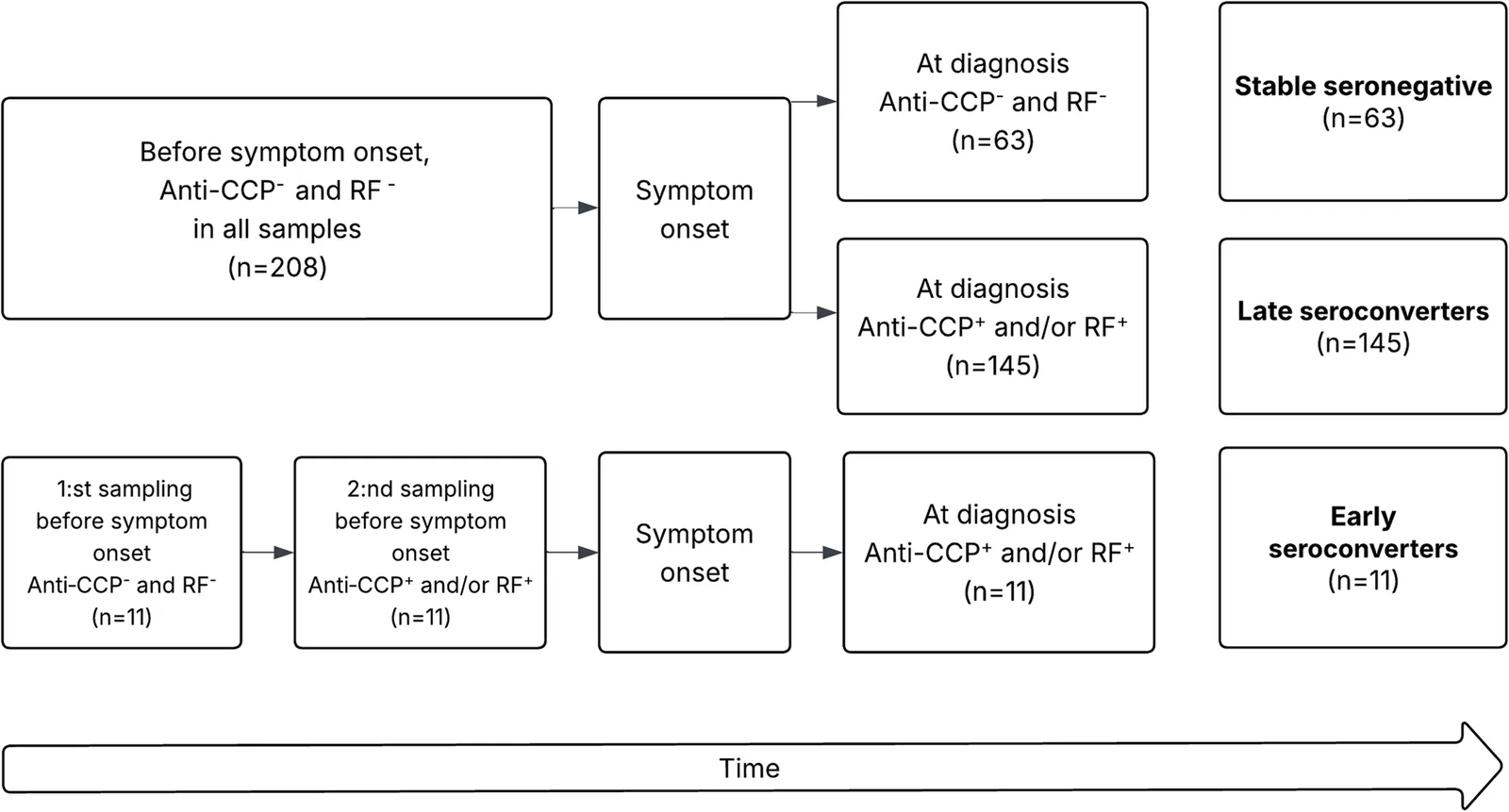
A flow chart depicting the subgroups included in the GAM analysis and the number of pre-symptomatic individuals. Stratification was based on anti‑CCP2 and RF status in pre-symptomatic samples and at diagnosis. Individuals positive for either anti-CCP or RF during the pre-symptomatic phase were not included in the flow chart (*n* = 199). Data on anti-CCP and RF were missing for one pre-symptomatic individual

To explore how protein levels differ between seropositive and seronegative individuals, and to assess proteins which were associated with the seroconversion, pre-symptomatic individuals were divided into three subgroups based on their autoantibody status (RF and/or anti-CCP) in the pre-symptomatic samples and at diagnosis, i.e. those who were seronegative in the earliest pre-symptomatic sample but seropositive in samples closer to symptom onset (*n* = 11) (early seroconverters); those who were seronegative in the pre-symptomatic sample but seropositive at diagnosis (*n* = 145) (late seroconverters); and those who remained seronegative in both pre-symptomatic samples as well as at diagnosis (*n* = 63) (stable seronegative) (Fig. 1).

Individuals who were seropositive in both pre-symptomatic samples and at diagnosis (*n* = 199) were excluded from the generalized additive model (GAM) analysis. One pre-symptomatic individual was excluded from the GAM analysis due to outlying results and in one pre-symptomatic individual information about anti-CCP or RF was lacking.

To ensure that individuals classified as late seroconverters did not simply appear to seroconvert later because they were sampled earlier in the pre-symptomatic phase than the early seroconverters, we compared the time from first reported symptoms to the sampling date. The mean time to symptom onset did not differ significantly between late and early seroconverters (–5.9 vs. − 6.25 years, *p* = 0.967). This indicates that the observed late seroconversion is unlikely to be an artifact of earlier sampling and instead reflects true differences in the timing of autoantibody development.

### Protein analysis

The levels of inflammation-related proteins were analysed with a commercially available proximity extension assay, Olink inflammation panel (Olink Proteomics) [18]. Initially, 92 proteins were measured; following quality control procedures recommended by the manufacturer, 77 proteins were retained for downstream analysis showing < 25% missing values. Protein concentrations are reported as Normalized Protein eXpression (NPX) values, which represent arbitrary units on a log2 scale normalized across samples.

### Antibody analysis

Samples from pre-symptomatic individuals, early RA patients and population controls were analysed for RF (Ogentec Diagnostika GmbH, Mainz, Germany) and anti-CCP2 (Eurodiagnostica, Malmö, Sweden). The anti-CCP2 analysis was performed according to the manufacturer’s instructions.

### Analysis of HLA

HLA-DRB1 genotyping was performed using polymerase chain reaction sequence-specific primers from DR low-resolution kit and DRB1*04 subtyping kit (Olerup SSP AB, Saltsjöbaden, Sweden). The HLA-SE genes were defined as 0401/0404/0405/0408/0101.

### Statistical analysis

All statistical analyses were performed using R statistical software version 4.3.2 [19]. Chi-square test was used for comparison of categorical values and Fisher’s exact test when appropriate. For comparison of continuous data for unrelated samples not normally distributed, the Mann-Whitney U-test or Kruskal-Wallis test were used. Generalized additive models (GAM) were used to compare differences in dynamics of the proteins over time to symptom onset, between different subgroups. For individuals with multiple samples, only the sample closest in time to symptom onset was included for each patient, if not specified otherwise. Generalized linear mixed models with logistic regression were used to analyse repeated measurements, treating each individual as a random effect, while protein concentration and time to symptom onset were included as fixed effects. In the generalized linear mixed effects models using autoantibody status and protein levels; in order to achieve convergence in the models an “all_fit” function from the lme4 R package was used and a converging optimizer was chosen for all proteins [20]. Odds ratios (OR) were calculated with 95% confidence intervals (95% CI). All p-values were two-sided and p-values less than or equal to 0.05 were considered statistically significant. To correct for multiple comparisons the Benjamini-Hochberg procedure was used for p-value correction [21]. Adjusted p-values (q-values, [P_corr_]) were calculated to control the false discovery rate (FDR), and results with q < 0.05 were considered statistically significant.

### Network enrichment analysis

To explore which biological pathways are associated with seroconversion, network enrichment analysis (NEA) was performed [22]. Altered gene sets (AGS) were identified by logistic regression analyses as described above. Proteins with unadjusted p-values 0.05 or less were included in the AGSs. Networks from STRING 12.0 [23] and Pathway Commons v.12 [24] were merged and used for the analysis. The merged network had in total 1.7 million links of which around 300 000 were shared between STRING and Pathway Commons. For the functional gene sets (FGS) pathways from Reactome [25] and BioCarta were used [26]. Compared to traditional overrepresentation analysis of pathway enrichment, NEA accounts for edges between the gene or protein nodes of AGS and FGS as well as the individual node degrees. Furthermore, to mitigate the bias introduced by the inclusion of inflammation-related proteins in the assay, we followed the approach of differential enrichment using a random permutation test as previously described [27].

## Results

### Difference in protein levels between pre-symptomatic individuals, early RA patients and controls

Levels of 77 inflammatory related proteins in plasma were compared between the different subject groups. Comparing pre-symptomatic individuals and controls, four proteins were significantly increased in samples from the pre-symptomatic period (MMP10, IL6, CCL19 and IL10RB, *p* < 0.05 for all) (Supplementary Table 1). Between pre-symptomatic individuals and early RA patients 62 proteins were significantly altered with S100A12, IL6 and TNFSF14 showing the strongest significance (P_corr_ <0.0001, for all) (Supplementary Table 1). Comparing samples from early RA patients and controls 54 proteins were significantly altered with: IL6, S100A12, CCL7 and TNFSF14 showing the strongest significance (P_corr_ <0.05, for all) (Supplementary Table 1).

### Difference in protein levels over time during the pre-symptomatic period

To identify proteins with significant relationship to time until symptom onset, a generalized additive model (GAM) was applied for each protein using the sample closest to symptom onset for all pre-symptomatic individuals. Eleven proteins showed significant relationships to time until symptom onset, CXCL9 (P_corr_ <0.05) and AXIN1, IL20RA, FGF23, LIFR, CXCL5, SIRT2, IL12B, CXCL10, DNER and TNFRSF9 (*p* < 0.05 and P_corr_ NS, for all) (data not shown).

In the five-year period closest to symptom onset a trend of increasing levels was found for TNFRSF9, CXCL10, IL12B, CXCL9, FGF23, and LIFR, whereas DNER, SIRT2, CXCL5, IL20RA, and AXIN1 showed a trend of decreasing levels (see Supplementary Fig. 1).

Pre-symptomatic individuals were compared with controls by stratifying the samples into quartiles based on the time from sampling to symptom onset. In the first quartile (-22 – -6.84 years before symptom onset, *n* = 104), one protein was significantly increased: MMP10 (*p* < 0.05). In the second quartile (-6.84 – -4.35 years, *n* = 105), EIF4EBP1, SULT1A1, STAMBP and SIRT2 were increased, and IL12B was decreased (*p* < 0.05). In the third quartile (-4.35 – -2.35 years, *n* = 105), CCL19, MMP10 and CDCP1 were increased compared to controls (*p* < 0.05). In the fourth quartile (-2.35 – -0.01 years, *n* = 105), 11 proteins were significantly altered, with IL6 and CCL19 showing the strongest significance: IL6, CCL19 (*p* < 0.01 for both), IL10RB, TNFRSF9, CCL7, CDCP1, CCL20, VEGFA, FLT3LG and CD5 were increased (*p* < 0.05), except for DNER, which decreased (*p* < 0.05) (Supplementary Table 2).

### Protein levels and autoantibody status during the pre-symptomatic period

Using simple logistic regression, we compared protein levels between seropositive and seronegative pre-symptomatic individuals (one sample per individual, closest to symptom onset), adjusting for time to symptom onset. Eleven proteins differed significantly between the two groups, namely TNFRSF9 (OR 3.37 95%CI 2.89–3.91, *p* < 0.001) and S100A12 (OR 1.71 95%CI 1.43–1.97, *p* < 0.001). The remaining significant proteins were CD5, CD6, CCL7, IL12B, CXCL9, CDCP1, IL6, CCL19, CXCL10 (OR between 1.3 and 2.1 *p* < 0.05, for all) (Table 2).

**Table 2:** Proteins analysed in pre-symptomatic individuals with a significant association to autoantibody status (anti-CCP+ and/ or RF+) using either a univariable logistic regression or a generalized linear mixed model adjusted for time to symptom onset

		Logistic regression model			General linear mixed model		
		(*n* = 418)^†^			(*n* = 472)^‡^		
Time to symptom, years (median [IQR])		-4.35 [-6.84, -2.35]			-4.69[-7.37,-2.50]		
Protein	Gene name	Odds ratio [95% CI]	*P* value	*P*_corr_^§^	Odds ratio [95% CI]	*P* value	*P*_corr_^§^
Tumor necrosis factor receptor superfamily member 9	TNFRSF9	3.4 [2.04, 5.66]	0.000002	0.00019	3.8 [2.09, 6.91]		0.0003
T-cell surface glycoprotein CD5	CD5	2.1 [1.19, 3.71]	0.012	0.14	2.4 [1.26, 4.48]	0.0073	0.080
T-cell differentiation antigen CD6	CD6	1.7 [1.14, 2.53]	0.0074	0.14	1.8 [1.13, 2.71]	0.012	0.099
Protein S100-A12	S100A12	1.7 [1.30, 2.22]	0.00011	0.0043	1.9 [1.35, 2.55]	0.00013	0.0024
C-C motif chemokine 7	CCL7	1.6 [1.15, 2.23]	0.0082	0.14	1.7 [1.16, 2.40]	0.0060	0.077
Interleukin-18 receptor 1	IL18R1	1.5 [0.91, 2.47]	0.099	0.51	1.8 [1.04, 2.95]	0.035	0.21
Interleukin-12 subunit beta	IL12B	1.5 [1.13, 2.00]	0.013	0.14	1.5 [1.08, 2.12]	0.016	0.11
C-X-C motif chemokine 9	CXCL9	1.4 [1.07, 1.83]	0.0091	0.14	1.5 [1.13, 1.99]	0.0053	0.077
CUB domain-containing protein 1	CDCP1	1.4 [1.01, 2.00]	0.043	0.30	1.5 [1.04, 2.18]	0.030	0.19
Interleukin-6	IL6	1.3 [1.03, 1.64]	0.014	0.14	1.3 [1.06, 1.65]	0.013	0.099
C-C motif chemokine 19	CCL19	1.3 [1.01, 1.67]	0.037	0.28	1.3 [1.01, 1.72]	0.040	0.22
C-X-C motif chemokine 10	CXCL10	1.3 [1.03, 1.64]	0.020	0.17	1.4 [1.08, 1.74]	0.010	0.099

^†^211 seronegative and 207 seropositive pre-symptomatic individuals. 1 sample missing RF and/or CCP information. Only included 1 sample per pre-symptomatic individual closest to symptom onset

^‡^245 seronegative and 227 seropositive samples from pre-symptomatic individuals. Including 55 with repeated samples with 2 samples each

^§^*p*-values adjusted for multiple comparisons using the Benjamini–Hochberg procedure

To strengthen the robustness of these findings, we additionally included the 55 pre-symptomatic individuals with repeated pre-symptomatic sampling. Binary autoantibody status at each sampling occasion was used as the outcome variable in a generalized linear mixed model (GLMM), which accounted for the non-independence of repeated samples from the same individual while adjusting for time to symptom onset. Of the 77 proteins, 12 proteins were significantly associated with autoantibody positivity namely, TNFRSF9 (OR 3.80 95%CI 1.45–6.15, *p* < 0.001), S100A12, (OR 1.86 95%CI 1.27–2.45, *p* < 0.001), as well as CD5, CD6, CCL7, IL12B, CXCL9, CDCP1, IL6, CCL19, CXCL10, IL18R1, (OR between 1.3 and 2.1, *p* < 0.05). S100A12 and TNFRSF9 also remained significant after correction for multiple comparisons (P_corr_<0.05) (Table 2). Two proteins (VEGFA, STAMBP) were excluded from the analysis due to non-convergence in the models.

To investigate how seropositivity influences the longitudinal dynamics of inflammation-related proteins, generalized additive models (GAMs) were constructed separately for each of the three groups (early seroconverters, late seroconverters, and stable seronegative cases). For each protein, concentration levels were modelled as a function of time stratified for subsets by seroconversion subgroup.

Six proteins showed a significant relation to time in the early seroconverters subgroup (GDNF, CCL2, CXCL11, FGF21, CXCL10, TNFRSF9, *p* < 0.05 for all, data not shown). In the late seroconverters subgroup, 11 proteins showed significance with time (CDCP1, IL20RA, MMP1, FGF23, CCL25, CCL8, CCL7, GDNF, TNFRSF11B, CST5, IFNG, *p* < 0.05 for all, data not shown). In the stable seronegative subgroup three proteins showed significant relationship to time (CXCL9, SLAMF1, FGF23, *p* < 0.05 for all, data not shown).

**Fig. 2:**
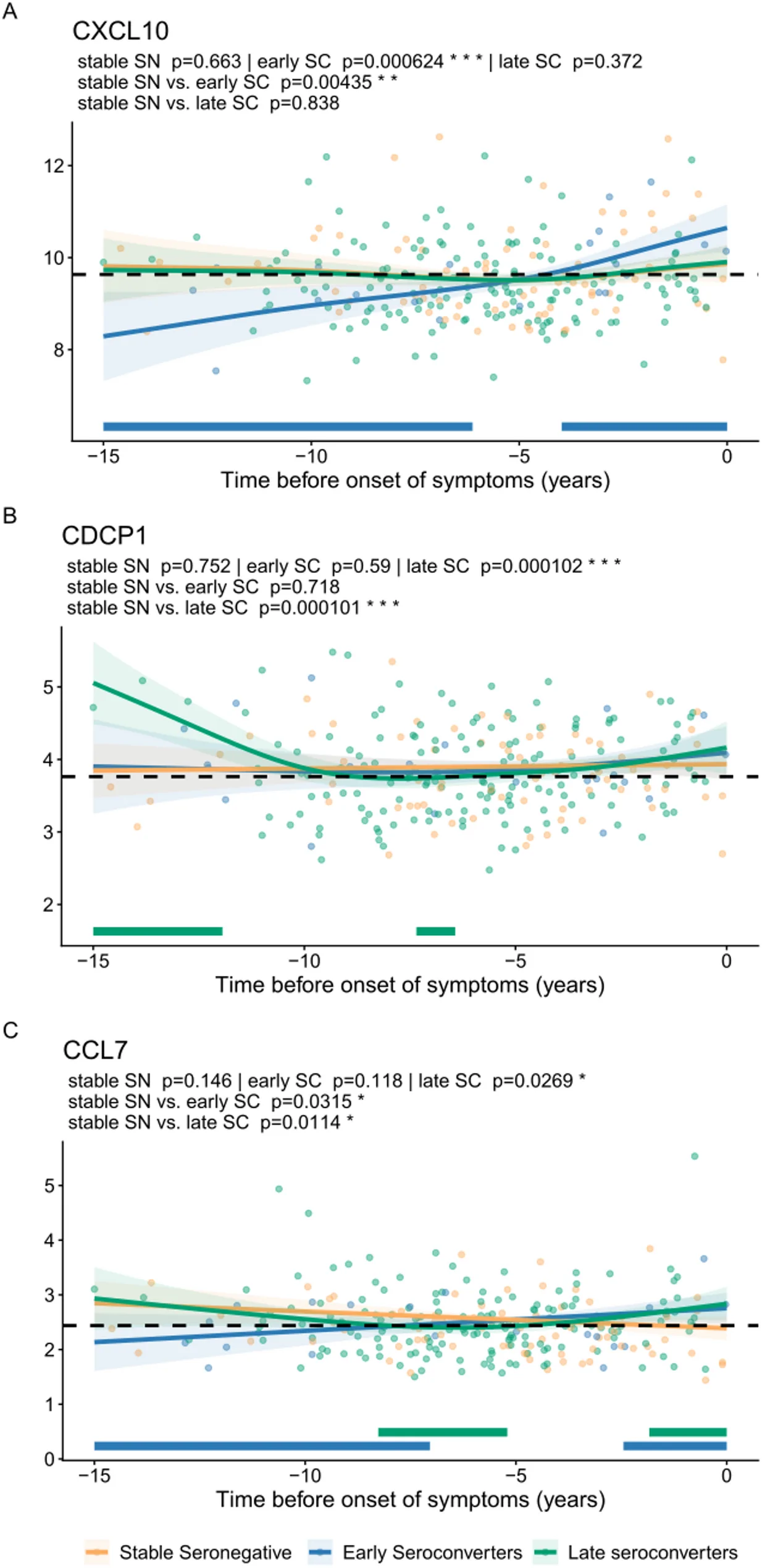
Generalized additive models (GAMs) illustrating protein levels (NPX) over time before onset of symptoms in each subgroup for (**A**) CXCL10, **B** CDCP1 and **C** CCL7. The first p-value indicates the significance of the GAM fit for protein concentration as a function of time for each curve (i.e. stable seronegative [SN], early and late seroconverters [SC]). The second p-value reflects the statistical difference between the stable SN (reference) curve and each of the two SC subgroups. The horizontal bar along the x-axis denotes time intervals where each model differs significantly from the reference curve (*p* < 0.05). The dashed line represents the median of the population controls. Statistical significance is indicated as follows: *p* < 0.05 (*), *p* < 0.01 (**), *p* < 0.001 (***)

To compare the models, the stable seronegative group was chosen as the reference model and compared to the seroconverter groups (i.e. early seroconverters and late seroconverters, respectively). Nine proteins differed significantly from the reference model (CCL7, GDNF, CDCP1, TNFRSF11B, FGF23, FGF21, CXCL10, IFNG, FGF19, *p* < 0.05 for all). The proteins that fulfilled the conditions of being both significant in the logistic regression analysis and differing significantly from the stable seronegative group in the GAM analysis were CXCL10, CDCP1, CCL7 (Fig. 2; Table 2).

No significant differences were observed between the early and late seroconverter groups regarding history of smoking or HLA-SE carrier status (*p* = 0.63 and *p* = 0.67, respectively).

**Fig. 3:**
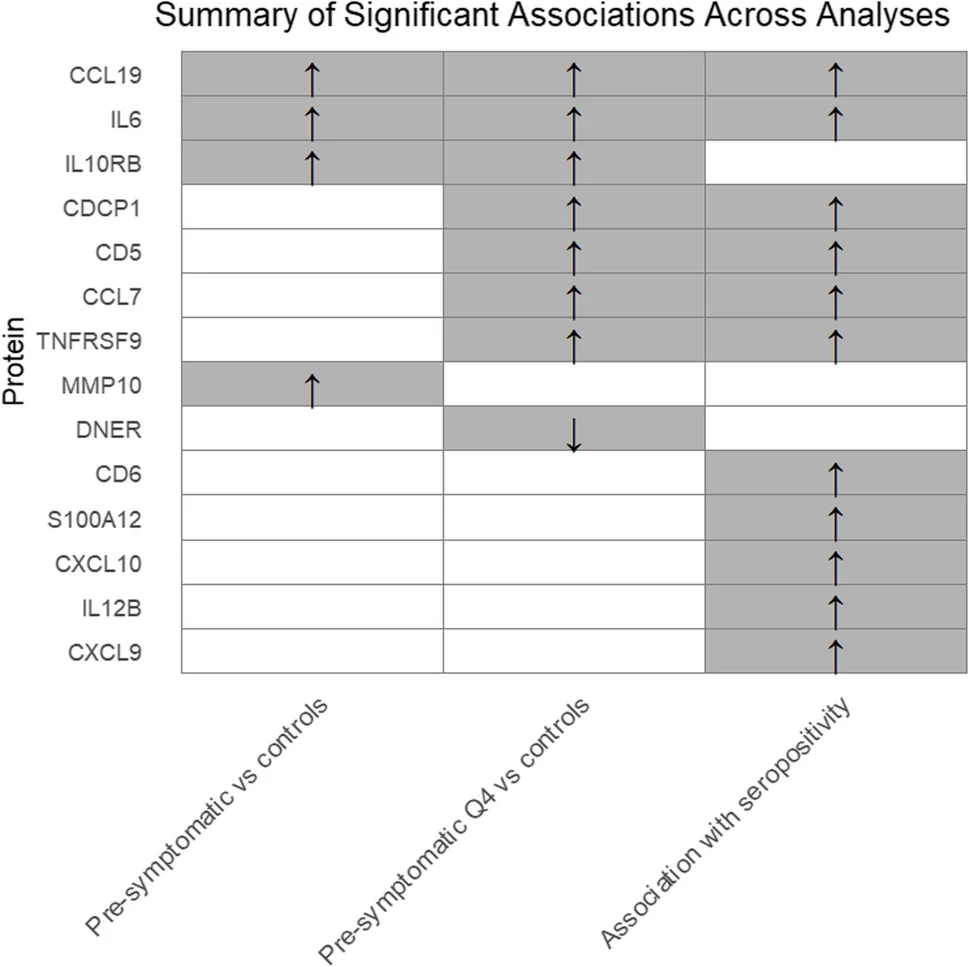
Illustrated summary of the associations between protein levels measured using Olink Inflammation panel in pre‑symptomatic individuals across different comparisons. **A** Pre-symptomatic individuals versus controls analysed using the Mann–Whitney U test. **B** Pre-symptomatic individuals sampled in the quartile closest to symptom onset (Q4) versus controls. **C** Seropositive versus seronegative individuals during the pre-symptomatic period using logistic regression analysis

To summarize, during the pre-symptomatic phase, a limited and recurring subset of proteins exhibited altered concentrations compared with controls and were also associated with seropositivity, either demonstrating progressive changes during the pre-dating time or relating to antibody presence (Fig. 3).

### Network enrichment analysis of proteins related to seroconversion

**Fig. 4:**
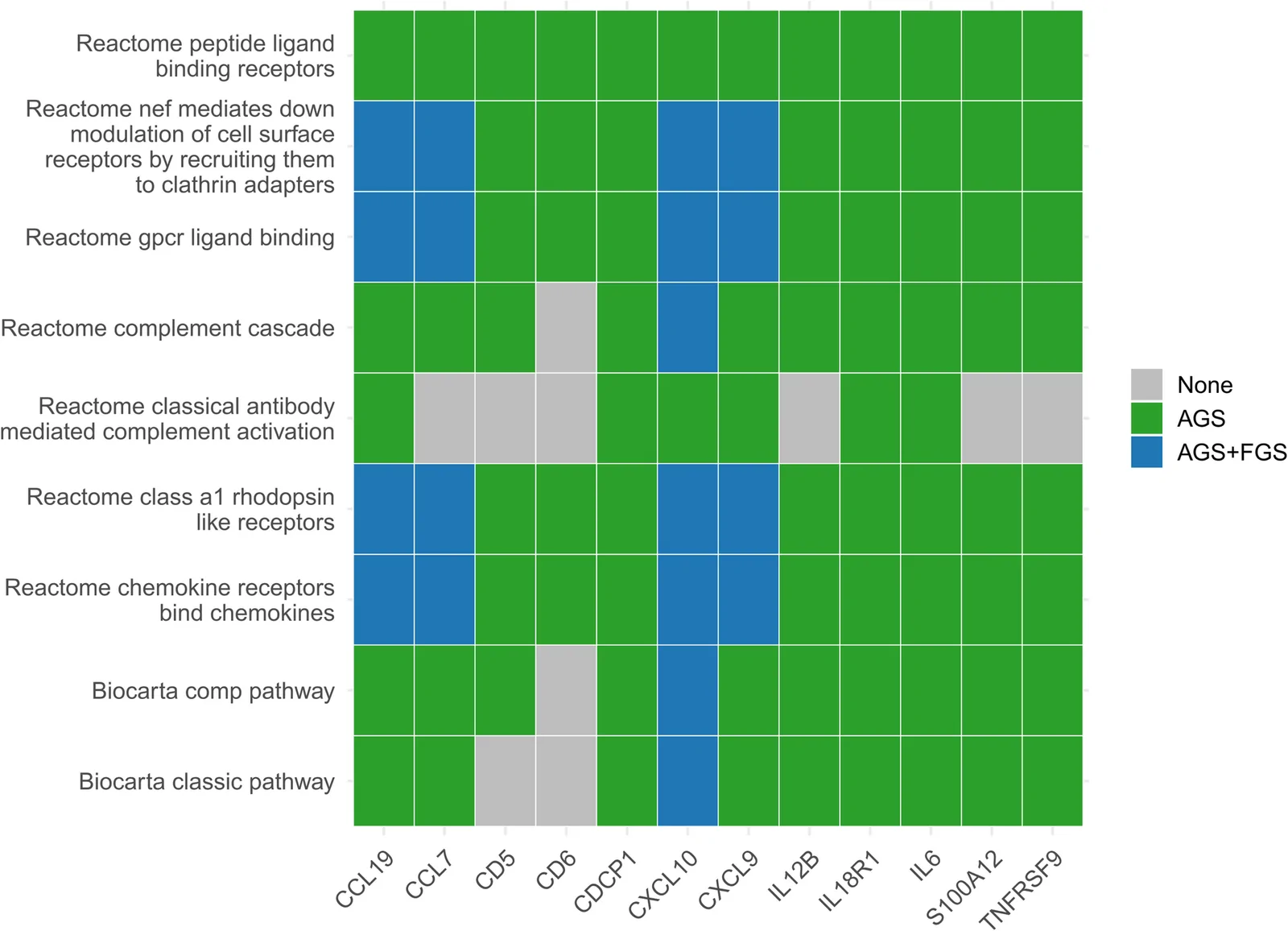
The 12 proteins associated with seropositivity in pre-symptomatic samples were defined as the altered gene set (AGS). Nine pathways were identified as significantly overrepresented (*p* < 0.05) after differential enrichment. Proteins labelled as AGS are only connected to pathways exclusively through network interactions (no direct membership in the functional gene set (FGS)), whereas AGS + FGS proteins are present in both the altered set and the functional gene sets, representing direct overlap

In a separate analysis, twelve proteins identified as significant through logistic regression or generalized linear mixed models (Table 2), examining the association between protein levels and seroconversion to anti-CCP and/or RF positivity, were subjected to network enrichment analysis using the Reactome and BioCarta databases. After adjusting for multiple comparisons, nine pathways were found to be significantly enriched. Four of these pathways represented GPCR ligand binding, Class A/1 Rhodopsin-like receptors, peptide ligand-binding receptors, and chemokine receptor activity. All were sub-pathways of a broader signal transduction pathway. Additionally, four pathways related to the classical complement cascade were consistently found using the Reactome and BioCarta datasets. A single pathway associated with Negative regulatory factor (Nef) downmodulation was also significantly enriched in Reactome. An overview of the proteins involved in each pathway is presented in Fig. 4.

## Discussion

In this study, we analysed pre-symptomatic samples from individuals who subsequently developed RA, comparing them with samples from early RA patients and healthy controls, and examined how inflammation-related proteins relate to seroconversion. Our findings support prior evidence of biological differences between seropositive and seronegative RA and show that these differences are detectable even before the onset of clinical symptoms.

Three proteins – CXCL10, CCL7 and CDCP1 – were repeatedly identified as associated with seroconversion across the logistic regression analysis, GLMM, and GAM analyses, with levels increasing over time as symptom onset approached in seroconverters but not in stable seronegative individuals. CXCL10 was lower at early pre-symptomatic time points and rose progressively. It has previously been linked to seropositive RA and to T-cell migration into the synovium, and pharmacologic blockade of CXCL10 attenuates arthritis in animal models [28, 29]. CCL7 rose mainly from around two years before symptom onset in late seroconverters and has recently been implicated in macrophage polarisation via JAK2/STAT1 signalling, a mechanism proposed to exacerbate synovial inflammation [30]. CDCP1 has been linked to IL6 production and to CD6-dependent T-cell adhesion to synovial fibroblasts, and elevated synovial CDCP1 has been reported in RA compared with osteoarthritis [31, 32]. Together, these proteins point to T-cell recruitment and early innate/adaptive crosstalk as plausible contributors to the earliest phase of seroconversion.

The proteins that changed over time differed by defined subgroup. In early seroconverters, most were linked to T-cell activity (e.g., CCL2, CXCL11, CXCL10), consistent with a T-cell-driven process in this phase. TNFRSF9, secreted by regulatory T cells, may plausibly contribute to the longer interval between seroconversion and symptom onset seen in this group [33–35]. In late seroconverters, proteins related to bone remodelling, mineral metabolism and collagen degradation predominated (e.g., MMP1, FGF23, TNFRSF11B, CST5) [36–38], suggesting a proteomic signature more indicative of synovial engagement and potentially a faster transition from seroconversion to symptom onset in this group. These findings are hypothesis-generating and require validation.

Network enrichment analysis of the seropositivity-associated proteins highlighted pathways related to the complement system and to G-protein-coupled/chemokine receptors. Complement involvement is consistent with reports of systemic complement activation in RA and of complement gene expression correlating with disease activity in early RA [39, 40], and our data extend this to the transition from pre-symptomatic to clinically apparent seropositive RA. The chemokine receptor signal likely reflects the broader elevation of cytokines and chemokines in seropositive pre-symptomatic individuals described above.

In the GAM analysis, seropositive and seronegative trajectories began to diverge approximately five years before symptom onset, and the quartile analysis similarly showed the most pronounced differences from controls in the final five years before onset. This is consistent with the hypothesis that autoimmune escalation intensifies in this window [12].

Four proteins (MMP10, IL6, CCL19, IL10RB) were higher in pre-symptomatic individuals than controls, though this did not survive correction for multiple testing. The modest control group size limits the power of these findings. MMP10 and IL6 were previously reported as elevated in pre-symptomatic individuals by our group although using different assay platforms [27, 41], supporting the plausibility of this observation despite limited power in the present study. IL6 is an established therapeutic target in RA and has been linked, together with ACPA positivity, to arthritis risk in arthralgia cohorts [42, 43] and MMP10 has been implicated in tissue destruction in RA [44]. CCL19 may promote immune cell trafficking and lymphoid structure formation via CCR7, and IL10RB, part of the IL-10 receptor complex, could plausibly modulate tolerance breakdown. Increased IL-10 has previously been associated with the pre-symptomatic period and with ACPA epitope spreading [11, 41].

Several limitations should be noted. Symptom onset was self-reported, introducing possible recall bias. The subgroups with repeated pre-symptomatic sampling and the control group were both relatively small, limiting statistical power, and further sample collection occurred opportunistically (in connection with mammography screening and population-based studies) rather than on a standardised schedule, thus timing relative to symptom onset varied. The findings have not yet been validated in an independent cohort. Early RA patients were classified using the 1987 ACR criteria, and data required for classification by the 2010 ACR/EULAR criteria are not systematically available for this cohort collected between 1996 and 2016. It is possible that findings could differ under current classification standards. The network enrichment analysis was restricted to the proteins associated with seropositivity and proteins relatively enriched in the seronegative group were not modelled as a separate gene set. The study is also limited to the predefined, antibody-dependent protein panel available on the Olink platform. Results were not validated using an orthogonal method, and, as with any antibody-based assay, PEA performance depends on antibody specificity and affinity and is subject to dynamic range constraints that may limit detection of very high- or low-abundance proteins. Balancing this, the cohort is among the largest available with sample collection predating symptom onset of RA, in a well-characterised, population-based, clinically verified early-RA registry.

In summary, CCL7, CXCL10 and CDCP1 are associated with seropositivity already in the pre-symptomatic period, supporting the view that seropositive and seronegative RA are biologically distinct from the earliest detectable stages, with immune dysregulation evident from approximately five years before symptom onset and involving chemokine receptor and complement pathways in particular. Further studies are needed to validate these observations.

## Supplementary Information

Supplementary Material 1. Supplementary Table 2. Protein concentrations in pre-symptomatic individuals were compared with controls, with pre-symptomatic samples stratified into four quartiles based on time to symptom onset. Statistical comparisons were performed using Mann-Whitney U tests for each quartile versus the control group. Unadjusted p-values and Benjamini-Hochberg–adjusted p-values are reported to account for multiple testing.

Supplementary Material 2. Supplementary Table 1. Protein concentrations across three study groups were compared using nonparametric statistical tests. A Kruskal-Wallis test was used to assess overall differences among pre-symptomatic individuals, early RA patients, and controls. Pairwise comparisons were performed using Mann-Whitney U tests for the following contrasts: pre-symptomatic vs. patient, control vs. patient, and pre-symptomatic vs. control. Unadjusted p-values are shown in the first column, and Benjamini-Hochberg–adjusted p-values are shown in the second column to account for multiple comparisons.

Supplementary Material 3. Supplementary Fig. 1. Generalized additive models (GAM) illustrating NPX concentrations as a function of time, with 95% confidence intervals shown. The 11 proteins with a statistically significant association with time (*p* < 0.05) are listed in this supplementary file.

## Data Availability

The datasets generated and/or analyzed during the current study are not publicly available due to the potential risk of participant re‑identification, even after data anonymization.
