# Supplementary material for: Temporal dynamics of inflammatory proteins in the pre-symptomatic phase of rheumatoid arthritis related to seroconversion
Source: Arthritis Res Ther. 2026 Oct 6;28:178. doi: 10.1186/s13075-026-03917-4 (PMC13644144; doi:10.1186/s13075-026-03917-4)
Supplement: Supplementary file 1 [file 13075_2026_3917_MOESM3_ESM.docx]

Supplementary figure 1. General additive models (GAM) illustrating NPX concentrations as a function of time, with 95% confidence intervals shown. The 11 proteins with a statistically significant association with time ($p < 0.05$) are listed in this supplementary file.

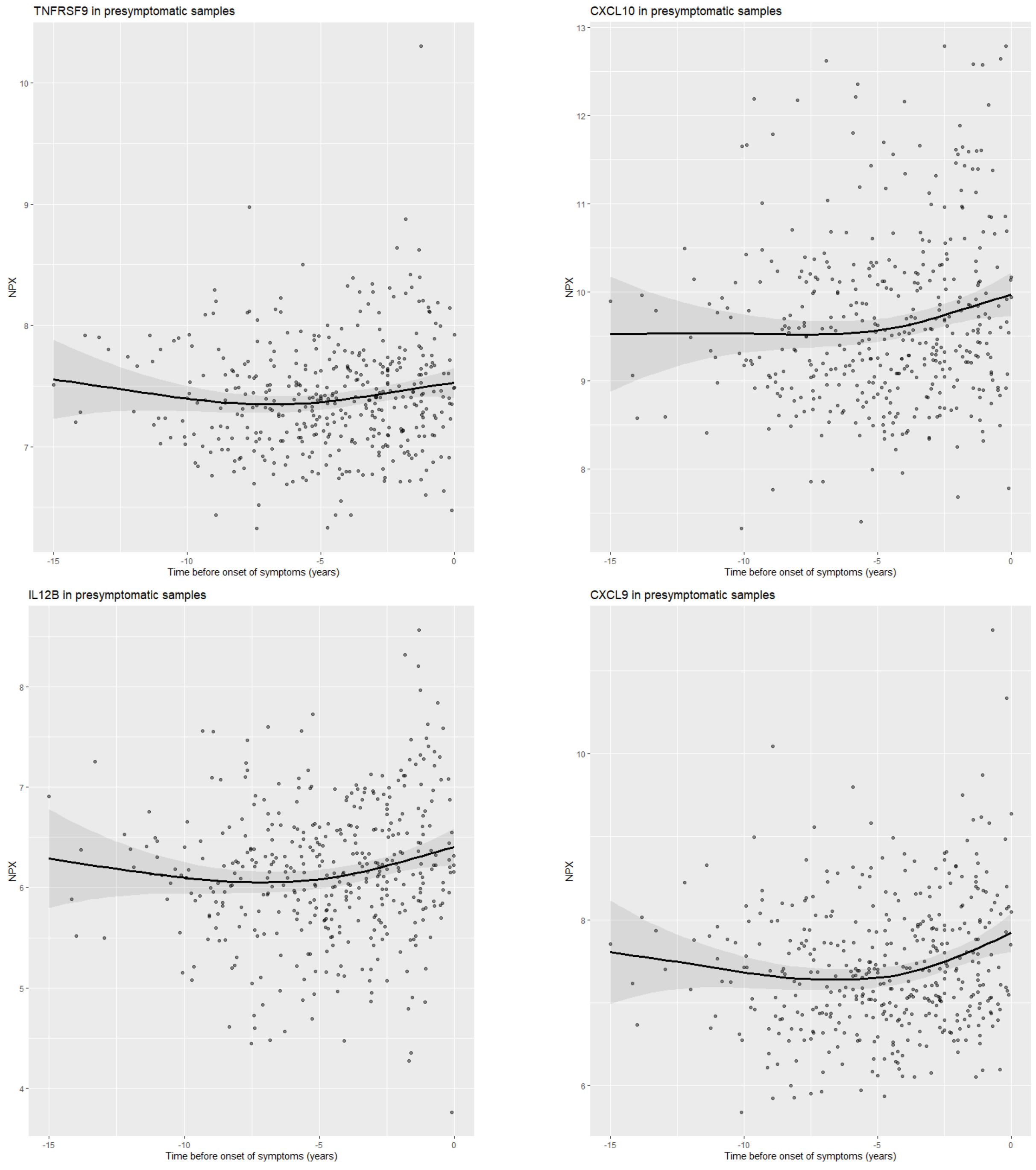

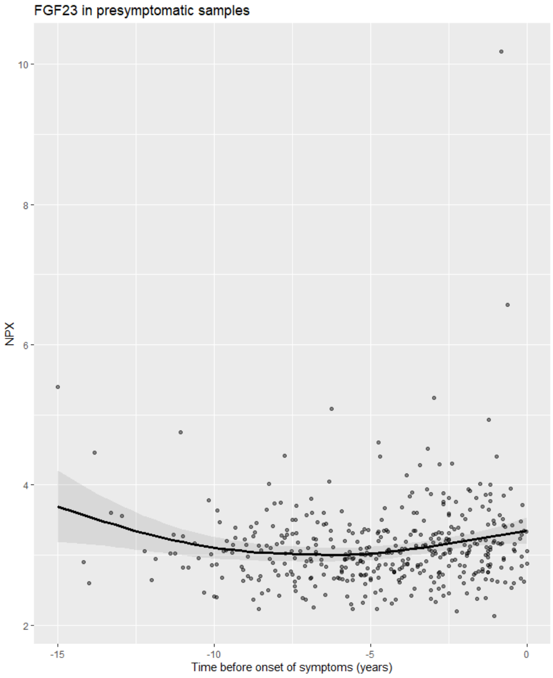
FGF23 in presymptomatic samples
NPX
10
8
6
4
2
-15
-10
-5
0
Time before onset of symptoms (years)

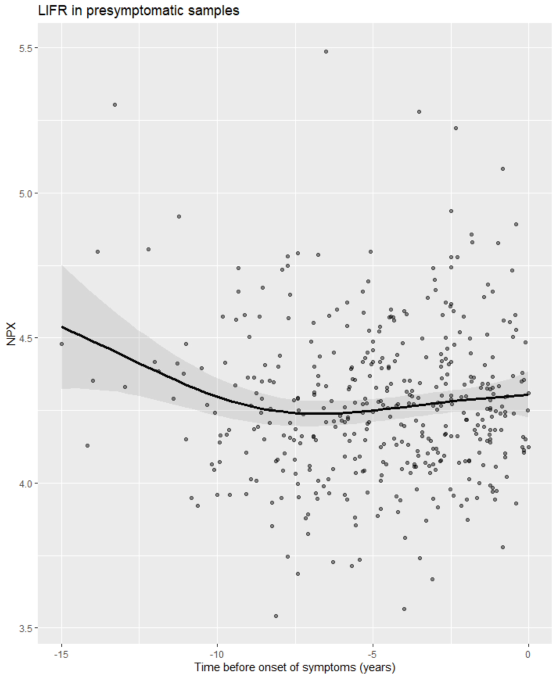
LIFR in presymptomatic samples
NPX
5.5
5.0
4.5
4.0
3.5
-15
-10
-5
0
Time before onset of symptoms (years)

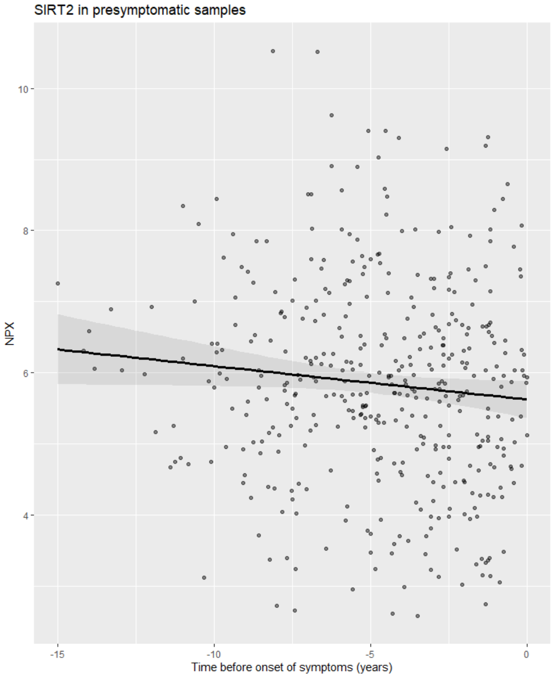
SIRT2 in presymptomatic samples
NPX
10
8
6
4
-15
-10
-5
0
Time before onset of symptoms (years)

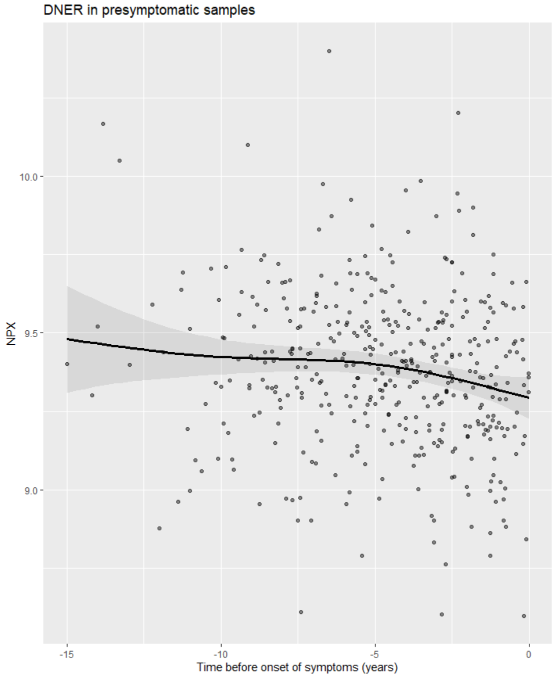
DNER in presymptomatic samples
NPX
10.0
9.5
9.0
-15
-10
-5
0
Time before onset of symptoms (years)

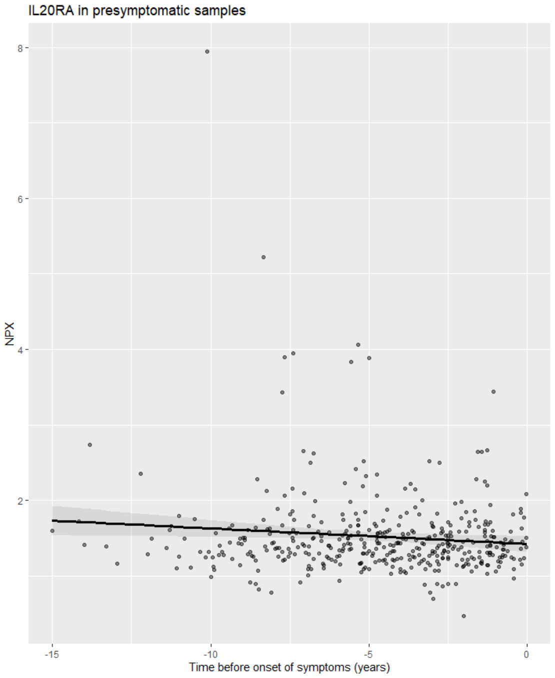

IL20RA in presymptomatic samples
NPX
Time before onset of symptoms (years)

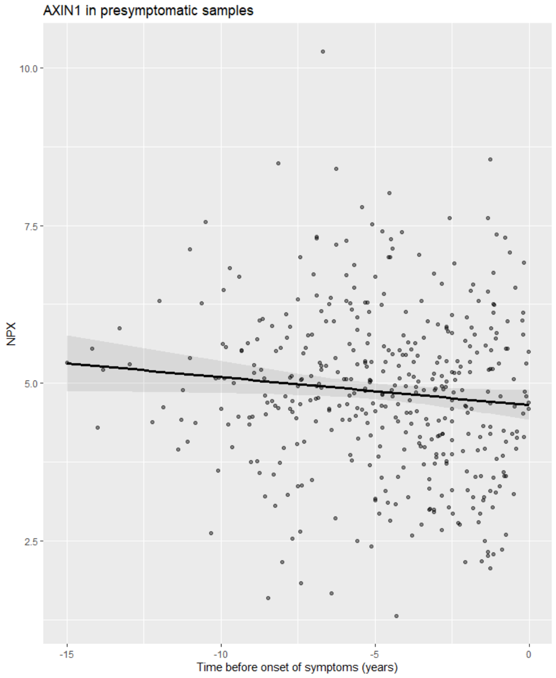

AXIN1 in presymptomatic samples
NPX
Time before onset of symptoms (years)

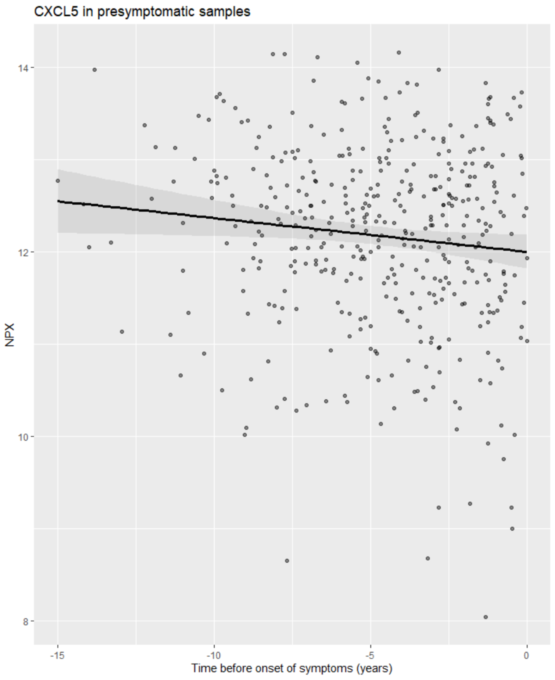

CXCL5 in presymptomatic samples
NPX
Time before onset of symptoms (years)
