# Supplementary material for: Temporal dynamics of inflammatory proteins in the pre-symptomatic phase of rheumatoid arthritis related to seroconversion
Source: Arthritis Res Ther. 2026 Oct 6;28:178. doi: 10.1186/s13075-026-03917-4 (PMC13644144; doi:10.1186/s13075-026-03917-4)
Supplement: Supplementary file 1 [file 13075_2026_3917_MOESM1_ESM.docx]

**Supplementary table 2.** Protein levels in pre-symptomatic individuals were compared with controls, with pre-symptomatic samples stratified into four quartiles based on time to symptom onset. Statistical comparisons were performed using Mann-Whitney U tests for each quartile versus the control group. Unadjusted p-values and Benjamini-Hochberg–adjusted p-values are reported to account for multiple testing.

|  |  | **Time to symptom onset quartiles compared to controls (n=49) (years)** | | | | | | | |
| --- | --- | --- | --- | --- | --- | --- | --- | --- | --- |
| **Protein name** | **Symbol** | **(-22,-6.86]**  **(n=104)** | | **(-6.86,-4.36]**  **(n=105)** | | **(-4.36,-2.36]**  **(n=105)** | | **(-2.36, 0.0]**  **(n=105)** | |
|  |  | **P-value** | **P-value_corr_** | **P-value** | **P-value_corr_** | **P-value** | **P-value_corr_** | **P-value** | **P-value_corr_** |
| Stromelysin-2 | MMP10 | **<0.05** | n.s. | n.s. | n.s. | **<0.05** | n.s. | n.s. | n.s. |
| Sulfotransferase 1A1 | SULT1A1 | n.s. | n.s. | **<0.05** | n.s. | n.s. | n.s. | n.s. | n.s. |
| STAM-binding protein | STAMBP | n.s. | n.s. | **<0.05** | n.s. | n.s. | n.s. | n.s. | n.s. |
| NAD-dependent protein deacetylase sirtuin-2 | SIRT2 | n.s. | n.s. | **<0.05** | n.s. | n.s. | n.s. | n.s. | n.s. |
| Interleukin-12 subunit beta | IL12B | n.s. | n.s. | **<0.05** | n.s. | n.s. | n.s. | n.s. | n.s. |
| Eukaryotic translation initiation factor 4E-binding protein 1 | EIF4EBP1 | n.s. | n.s. | **<0.05** | n.s. | n.s. | n.s. | n.s. | n.s. |
| CUB domain-containing protein 1 | CDCP1 | n.s. | n.s. | n.s. | n.s. | **<0.05** | n.s. | **<0.05** | n.s. |
| C-C motif chemokine 19 | CCL19 | n.s. | n.s. | n.s. | n.s. | **<0.05** | n.s. | **<0.01** | n.s. |
| Interleukin-6 | IL6 | n.s. | n.s. | n.s. | n.s. | n.s. | n.s. | **<0.01** | n.s. |
| Vascular endothelial growth factor A | VEGFA | n.s. | n.s. | n.s. | n.s. | n.s. | n.s. | **<0.05** | n.s. |
| Tumor necrosis factor receptor superfamily member 9 | TNFRSF9 | n.s. | n.s. | n.s. | n.s. | n.s. | n.s. | **<0.05** | n.s. |
| Interleukin-10 receptor subunit beta | IL10RB | n.s. | n.s. | n.s. | n.s. | n.s. | n.s. | **<0.05** | n.s. |
| Fms-related tyrosine kinase 3 ligand | FLT3LG | n.s. | n.s. | n.s. | n.s. | n.s. | n.s. | **<0.05** | n.s. |
| Delta and Notch-like epidermal growth factor-related receptor | DNER | n.s. | n.s. | n.s. | n.s. | n.s. | n.s. | **<0.05** | n.s. |
| C-C motif chemokine 7 | CCL7 | n.s. | n.s. | n.s. | n.s. | n.s. | n.s. | **<0.05** | n.s. |
| C-C motif chemokine 20 | CCL20 | n.s. | n.s. | n.s. | n.s. | n.s. | n.s. | **<0.05** | n.s. |
| Tumor necrosis factor ligand superfamily member 14 | TNFSF14 | n.s. | n.s. | n.s. | n.s. | n.s. | n.s. | n.s. | n.s. |
| Tumor necrosis factor ligand superfamily member 12 | TNFSF12 | n.s. | n.s. | n.s. | n.s. | n.s. | n.s. | n.s. | n.s. |
| Tumor necrosis factor ligand superfamily member 11 | TNFSF11 | n.s. | n.s. | n.s. | n.s. | n.s. | n.s. | n.s. | n.s. |
| Tumor necrosis factor ligand superfamily member 10 | TNFSF10 | n.s. | n.s. | n.s. | n.s. | n.s. | n.s. | n.s. | n.s. |
| Tumor necrosis factor receptor superfamily member 11B | TNFRSF11B | n.s. | n.s. | n.s. | n.s. | n.s. | n.s. | n.s. | n.s. |
| Tumor necrosis factor | TNF | n.s. | n.s. | n.s. | n.s. | n.s. | n.s. | n.s. | n.s. |
| Transforming growth factor beta-1 proprotein | TGFB1 | n.s. | n.s. | n.s. | n.s. | n.s. | n.s. | n.s. | n.s. |
| Protransforming growth factor alpha | TGFA | n.s. | n.s. | n.s. | n.s. | n.s. | n.s. | n.s. | n.s. |
| Signaling lymphocytic activation molecule | SLAMF1 | n.s. | n.s. | n.s. | n.s. | n.s. | n.s. | n.s. | n.s. |
| Protein S100-A12 | S100A12 | n.s. | n.s. | n.s. | n.s. | n.s. | n.s. | n.s. | n.s. |
| Urokinase-type plasminogen activator | PLAU | n.s. | n.s. | n.s. | n.s. | n.s. | n.s. | n.s. | n.s. |
| Oncostatin-M | OSM | n.s. | n.s. | n.s. | n.s. | n.s. | n.s. | n.s. | n.s. |
| Neurotrophin-3 | NTF3 | n.s. | n.s. | n.s. | n.s. | n.s. | n.s. | n.s. | n.s. |
| Interstitial collagenase | MMP1 | n.s. | n.s. | n.s. | n.s. | n.s. | n.s. | n.s. | n.s. |
| Lymphotoxin-alpha | LTA | n.s. | n.s. | n.s. | n.s. | n.s. | n.s. | n.s. | n.s. |
| Leukemia inhibitory factor receptor | LIFR | n.s. | n.s. | n.s. | n.s. | n.s. | n.s. | n.s. | n.s. |
| Kit ligand | KITLG | n.s. | n.s. | n.s. | n.s. | n.s. | n.s. | n.s. | n.s. |
| Interleukin-7 | IL7 | n.s. | n.s. | n.s. | n.s. | n.s. | n.s. | n.s. | n.s. |
| Interleukin-20 receptor subunit alpha | IL20RA | n.s. | n.s. | n.s. | n.s. | n.s. | n.s. | n.s. | n.s. |
| Interleukin-18 receptor 1 | IL18R1 | n.s. | n.s. | n.s. | n.s. | n.s. | n.s. | n.s. | n.s. |
| Interleukin-18 | IL18 | n.s. | n.s. | n.s. | n.s. | n.s. | n.s. | n.s. | n.s. |
| Interleukin-17C | IL17C | n.s. | n.s. | n.s. | n.s. | n.s. | n.s. | n.s. | n.s. |
| Interleukin-17A | IL17A | n.s. | n.s. | n.s. | n.s. | n.s. | n.s. | n.s. | n.s. |
| Interleukin-15 receptor subunit alpha | IL15RA | n.s. | n.s. | n.s. | n.s. | n.s. | n.s. | n.s. | n.s. |
| Interleukin-10 receptor subunit alpha | IL10RA | n.s. | n.s. | n.s. | n.s. | n.s. | n.s. | n.s. | n.s. |
| Interleukin-10 | IL10 | n.s. | n.s. | n.s. | n.s. | n.s. | n.s. | n.s. | n.s. |
| Interferon gamma | IFNG | n.s. | n.s. | n.s. | n.s. | n.s. | n.s. | n.s. | n.s. |
| Hepatocyte growth factor | HGF | n.s. | n.s. | n.s. | n.s. | n.s. | n.s. | n.s. | n.s. |
| Glial cell line-derived neurotrophic factor | GDNF | n.s. | n.s. | n.s. | n.s. | n.s. | n.s. | n.s. | n.s. |
| Fibroblast growth factor 5 | FGF5 | n.s. | n.s. | n.s. | n.s. | n.s. | n.s. | n.s. | n.s. |
| Fibroblast growth factor 23 | FGF23 | n.s. | n.s. | n.s. | n.s. | n.s. | n.s. | n.s. | n.s. |
| Fibroblast growth factor 21 | FGF21 | n.s. | n.s. | n.s. | n.s. | n.s. | n.s. | n.s. | n.s. |
| Fibroblast growth factor 19 | FGF19 | n.s. | n.s. | n.s. | n.s. | n.s. | n.s. | n.s. | n.s. |
| C-X-C motif chemokine 9 | CXCL9 | n.s. | n.s. | n.s. | n.s. | n.s. | n.s. | n.s. | n.s. |
| Interleukin-8 | CXCL8 | n.s. | n.s. | n.s. | n.s. | n.s. | n.s. | n.s. | n.s. |
| C-X-C motif chemokine 6 | CXCL6 | n.s. | n.s. | n.s. | n.s. | n.s. | n.s. | n.s. | n.s. |
| C-X-C motif chemokine 5 | CXCL5 | n.s. | n.s. | n.s. | n.s. | n.s. | n.s. | n.s. | n.s. |
| C-X-C motif chemokine 11 | CXCL11 | n.s. | n.s. | n.s. | n.s. | n.s. | n.s. | n.s. | n.s. |
| C-X-C motif chemokine 10 | CXCL10 | n.s. | n.s. | n.s. | n.s. | n.s. | n.s. | n.s. | n.s. |
| Growth-regulated alpha protein | CXCL1 | n.s. | n.s. | n.s. | n.s. | n.s. | n.s. | n.s. | n.s. |
| Fractalkine | CX3CL1 | n.s. | n.s. | n.s. | n.s. | n.s. | n.s. | n.s. | n.s. |
| Cystatin-D | CST5 | n.s. | n.s. | n.s. | n.s. | n.s. | n.s. | n.s. | n.s. |
| Macrophage colony-stimulating factor 1 | CSF1 | n.s. | n.s. | n.s. | n.s. | n.s. | n.s. | n.s. | n.s. |
| T-cell surface glycoprotein CD8 alpha chain | CD8A | n.s. | n.s. | n.s. | n.s. | n.s. | n.s. | n.s. | n.s. |
| T-cell differentiation antigen CD6 | CD6 | n.s. | n.s. | n.s. | n.s. | n.s. | n.s. | n.s. | n.s. |
| T-cell surface glycoprotein CD5 | CD5 | n.s. | n.s. | n.s. | n.s. | n.s. | n.s. | n.s. | n.s. |
| Tumor necrosis factor receptor superfamily member 5 | CD40 | n.s. | n.s. | n.s. | n.s. | n.s. | n.s. | n.s. | n.s. |
| Programmed cell death 1 ligand 1 | CD274 | n.s. | n.s. | n.s. | n.s. | n.s. | n.s. | n.s. | n.s. |
| Natural killer cell receptor 2B4 | CD244 | n.s. | n.s. | n.s. | n.s. | n.s. | n.s. | n.s. | n.s. |
| C-C motif chemokine 8 | CCL8 | n.s. | n.s. | n.s. | n.s. | n.s. | n.s. | n.s. | n.s. |
| C-C motif chemokine 4 | CCL4 | n.s. | n.s. | n.s. | n.s. | n.s. | n.s. | n.s. | n.s. |
| C-C motif chemokine 3 | CCL3 | n.s. | n.s. | n.s. | n.s. | n.s. | n.s. | n.s. | n.s. |
| C-C motif chemokine 28 | CCL28 | n.s. | n.s. | n.s. | n.s. | n.s. | n.s. | n.s. | n.s. |
| C-C motif chemokine 25 | CCL25 | n.s. | n.s. | n.s. | n.s. | n.s. | n.s. | n.s. | n.s. |
| C-C motif chemokine 23 | CCL23 | n.s. | n.s. | n.s. | n.s. | n.s. | n.s. | n.s. | n.s. |
| C-C motif chemokine 2 | CCL2 | n.s. | n.s. | n.s. | n.s. | n.s. | n.s. | n.s. | n.s. |
| C-C motif chemokine 13 | CCL13 | n.s. | n.s. | n.s. | n.s. | n.s. | n.s. | n.s. | n.s. |
| Eotaxin | CCL11 | n.s. | n.s. | n.s. | n.s. | n.s. | n.s. | n.s. | n.s. |
| Caspase-8 | CASP8 | n.s. | n.s. | n.s. | n.s. | n.s. | n.s. | n.s. | n.s. |
| Axin-1 | AXIN1 | n.s. | n.s. | n.s. | n.s. | n.s. | n.s. | n.s. | n.s. |
| Adenosine deaminase | ADA | n.s. | n.s. | n.s. | n.s. | n.s. | n.s. | n.s. | n.s. |
| Statistical analysis was performed using the Mann-Whitney U test for pairwise group comparisons. P-values were adjusted for multiple comparisons using the Benjamini-Hochberg procedure. | | | | | | | | | |
