# Supplementary material for: Temporal dynamics of inflammatory proteins in the pre-symptomatic phase of rheumatoid arthritis related to seroconversion
Source: Arthritis Res Ther. 2026 Oct 6;28:178. doi: 10.1186/s13075-026-03917-4 (PMC13644144; doi:10.1186/s13075-026-03917-4)
Supplement: Supplementary file 2 [file 13075_2026_3917_MOESM2_ESM.docx]

**Supplementary table 1.** Protein concentrations across pre-symptomatic individuals, controls and eRA patients were compared using nonparametric statistical tests. Pairwise comparisons were performed using Mann-Whitney U tests for the following contrasts: pre-symptomatic vs eRA patients, control vs eRA patients, and pre-symptomatic vs controls. Presented in descending order from left to right based on p-values.
Unadjusted p-values are shown in the first column, and Benjamini-Hochberg–adjusted p-values are shown in the second column to account for multiple comparisons**.**

|  | | **Protein concentrations (NPX)** | | | **Pre-symptomatic individuals vs. Controls^‡^** | | **Pre-symptomatic individuals  vs.Early RA Patients^‡^** | | **Early RA Patients vs.  Controls^‡^** | |
| --- | --- | --- | --- | --- | --- | --- | --- | --- | --- | --- |
| **Protein name** | **Symbol** | **Control  (median)** | **Pre-symptomatic  individuals  (median)** | **Early RA- Patients  (median)** | **P-value** | **P-value_corr_^§^** | **P-value** | **P-value_corr_^§^** | **P-value** | **P-value_corr_^§^** |
| Interleukin-6 | IL6 | 3.6 | 3.9 | 6.6 | **<0.05** | n.s. | **<0.001** | **<0.001** | **<0.001** | **<0.001** |
| C-C motif chemokine 19 | CCL19 | 9.2 | 9.3 | 10.0 | **<0.05** | n.s. | **<0.001** | **<0.001** | **<0.001** | **<0.001** |
| Interleukin-10 receptor subunit beta | IL10RB | 6.2 | 6.3 | 6.5 | **<0.05** | n.s. | **<0.001** | **<0.001** | **<0.001** | **<0.001** |
| Stromelysin-2 | MMP10 | 8.8 | 9.0 | 9.0 | **<0.05** | n.s. | n.s. | n.s. | **<0.01** | **<0.01** |
| Protein S100-A12 | S100A12 | 3.0 | 2.8 | 5.0 | n.s. | n.s. | **<0.001** | **<0.001** | **<0.001** | **<0.001** |
| C-C motif chemokine 7 | CCL7 | 2.4 | 2.5 | 3.6 | n.s. | n.s. | **<0.001** | **<0.001** | **<0.001** | **<0.001** |
| Tumor necrosis factor ligand superfamily member 14 | TNFSF14 | 4.4 | 4.5 | 5.6 | n.s. | n.s. | **<0.001** | **<0.001** | **<0.001** | **<0.001** |
| Vascular endothelial growth factor A | VEGFA | 10.7 | 10.8 | 11.4 | n.s. | n.s. | **<0.001** | **<0.001** | **<0.001** | **<0.001** |
| Macrophage colony-stimulating factor 1 | CSF1 | 10.2 | 10.3 | 10.6 | n.s. | n.s. | **<0.001** | **<0.001** | **<0.001** | **<0.001** |
| Hepatocyte growth factor | HGF | 8.7 | 8.8 | 9.3 | n.s. | n.s. | **<0.001** | **<0.001** | **<0.001** | **<0.001** |
| Transforming growth factor beta-1 proprotein | TGFB1 | 8.0 | 8.1 | 8.7 | n.s. | n.s. | **<0.001** | **<0.001** | **<0.001** | **<0.001** |
| C-C motif chemokine 23 | CCL23 | 10.0 | 10.1 | 10.7 | n.s. | n.s. | **<0.001** | **<0.001** | **<0.001** | **<0.001** |
| C-X-C motif chemokine 11 | CXCL11 | 8.4 | 8.4 | 9.5 | n.s. | n.s. | **<0.001** | **<0.001** | **<0.001** | **<0.001** |
| Glial cell line-derived neurotrophic factor | GDNF | 2.4 | 2.5 | 2.8 | n.s. | n.s. | **<0.001** | **<0.001** | **<0.001** | **<0.001** |
| Growth-regulated alpha protein | CXCL1 | 9.7 | 9.9 | 10.5 | n.s. | n.s. | **<0.001** | **<0.001** | **<0.001** | **<0.001** |
| Interleukin-7 | IL7 | 4.0 | 4.0 | 4.8 | n.s. | n.s. | **<0.001** | **<0.001** | **<0.001** | **<0.001** |
| Interleukin-10 | IL10 | 4.0 | 4.1 | 4.6 | n.s. | n.s. | **<0.001** | **<0.001** | **<0.001** | **<0.001** |
| Programmed cell death 1 ligand 1 | CD274 | 6.8 | 6.9 | 7.3 | n.s. | n.s. | **<0.001** | **<0.001** | **<0.001** | **<0.001** |
| Tumor necrosis factor receptor superfamily member 5 | CD40 | 11.9 | 12.0 | 12.5 | n.s. | n.s. | **<0.001** | **<0.001** | **<0.001** | **<0.001** |
| C-C motif chemokine 20 | CCL20 | 6.2 | 6.6 | 7.4 | n.s. | n.s. | **<0.001** | **<0.001** | **<0.001** | **<0.001** |
| C-X-C motif chemokine 5 | CXCL5 | 12.3 | 12.3 | 13.2 | n.s. | n.s. | **<0.001** | **<0.001** | **<0.001** | **<0.001** |
| CUB domain-containing protein 1 | CDCP1 | 3.6 | 3.9 | 4.5 | n.s. | n.s. | **<0.001** | **<0.001** | **<0.001** | **<0.001** |
| Eukaryotic translation initiation factor 4E-binding protein 1 | EIF4EBP1 | 7.9 | 8.4 | 10.0 | n.s. | n.s. | **<0.001** | **<0.001** | **<0.001** | **<0.001** |
| Interleukin-18 receptor 1 | IL18R1 | 8.7 | 8.7 | 9.1 | n.s. | n.s. | **<0.001** | **<0.001** | **<0.001** | **<0.001** |
| C-X-C motif chemokine 10 | CXCL10 | 9.6 | 9.5 | 10.8 | n.s. | n.s. | **<0.001** | **<0.001** | **<0.001** | **<0.001** |
| Caspase-8 | CASP8 | 3.9 | 4.2 | 5.2 | n.s. | n.s. | **<0.001** | **<0.001** | **<0.001** | **<0.001** |
| NAD-dependent protein deacetylase sirtuin-2 | SIRT2 | 5.7 | 5.8 | 7.8 | n.s. | n.s. | **<0.001** | **<0.001** | **<0.001** | **<0.001** |
| Interleukin-8 | CXCL8 | 5.3 | 5.3 | 5.9 | n.s. | n.s. | **<0.001** | **<0.001** | **<0.001** | **<0.001** |
| Oncostatin-M | OSM | 3.2 | 3.4 | 4.4 | n.s. | n.s. | **<0.001** | **<0.001** | **<0.001** | **<0.001** |
| C-C motif chemokine 13 | CCL13 | 13.7 | 13.8 | 14.5 | n.s. | n.s. | **<0.001** | **<0.001** | **<0.001** | **<0.001** |
| STAM-binding protein | STAMBP | 6.4 | 6.5 | 8.0 | n.s. | n.s. | **<0.001** | **<0.001** | **<0.001** | **<0.001** |
| C-X-C motif chemokine 9 | CXCL9 | 7.5 | 7.3 | 8.0 | n.s. | n.s. | **<0.001** | **<0.001** | **<0.001** | **<0.001** |
| Sulfotransferase 1A1 | SULT1A1 | 4.8 | 5.0 | 5.9 | n.s. | n.s. | **<0.001** | **<0.001** | **<0.001** | **<0.001** |
| Protransforming growth factor alpha | TGFA | 3.6 | 3.6 | 3.8 | n.s. | n.s. | **<0.001** | **<0.001** | **<0.001** | **<0.001** |
| Tumor necrosis factor receptor superfamily member 11B | TNFRSF11B | 10.3 | 10.4 | 10.6 | n.s. | n.s. | **<0.001** | **<0.001** | **<0.001** | **<0.001** |
| Interleukin-18 | IL18 | 9.0 | 9.0 | 9.6 | n.s. | n.s. | **<0.001** | **<0.001** | **<0.001** | **<0.001** |
| Axin-1 | AXIN1 | 4.8 | 4.9 | 6.0 | n.s. | n.s. | **<0.001** | **<0.001** | **<0.001** | **<0.001** |
| C-X-C motif chemokine 6 | CXCL6 | 9.3 | 9.0 | 10.0 | n.s. | n.s. | **<0.001** | **<0.001** | **<0.001** | **<0.001** |
| C-C motif chemokine 8 | CCL8 | 9.2 | 9.2 | 9.6 | n.s. | n.s. | **<0.001** | **<0.001** | **<0.001** | **<0.001** |
| Tumor necrosis factor receptor superfamily member 9 | TNFRSF9 | 7.2 | 7.4 | 7.6 | n.s. | n.s. | **<0.001** | **<0.001** | **<0.001** | **<0.001** |
| T-cell surface glycoprotein CD5 | CD5 | 5.5 | 5.6 | 5.7 | n.s. | n.s. | **<0.001** | **<0.001** | **<0.001** | **<0.001** |
| Natural killer cell receptor 2B4 | CD244 | 7.7 | 7.7 | 7.9 | n.s. | n.s. | **<0.001** | **<0.001** | **<0.001** | **<0.01** |
| Adenosine deaminase | ADA | 5.5 | 5.5 | 5.8 | n.s. | n.s. | **<0.001** | **<0.001** | **<0.001** | **<0.01** |
| Tumor necrosis factor | TNF | 3.7 | 3.7 | 4.1 | n.s. | n.s. | **<0.001** | **<0.001** | **<0.01** | **<0.01** |
| Signaling lymphocytic activation molecule | SLAMF1 | 2.0 | 2.0 | 2.1 | n.s. | n.s. | **<0.001** | **<0.001** | **<0.01** | **<0.01** |
| Interleukin-15 receptor subunit alpha | IL15RA | 2.1 | 2.1 | 2.2 | n.s. | n.s. | **<0.001** | **<0.001** | **<0.01** | **<0.01** |
| C-C motif chemokine 4 | CCL4 | 6.3 | 6.2 | 6.6 | n.s. | n.s. | **<0.001** | **<0.001** | **<0.01** | **<0.01** |
| C-C motif chemokine 28 | CCL28 | 2.5 | 2.5 | 2.7 | n.s. | n.s. | **<0.001** | **<0.001** | **<0.01** | **<0.01** |
| Interstitial collagenase | MMP1 | 11.0 | 11.0 | 11.5 | n.s. | n.s. | **<0.001** | **<0.001** | **<0.05** | **<0.05** |
| C-C motif chemokine 3 | CCL3 | 5.0 | 5.0 | 5.3 | n.s. | n.s. | **<0.001** | **<0.001** | **<0.05** | **<0.05** |
| Cystatin-D | CST5 | 5.6 | 5.5 | 5.8 | n.s. | n.s. | **<0.001** | **<0.001** | n.s. | n.s. |
| Interleukin-10 receptor subunit alpha | IL10RA | 1.5 | 1.5 | 1.7 | n.s. | n.s. | **<0.01** | **<0.01** | **<0.05** | **<0.05** |
| Interleukin-20 receptor subunit alpha | IL20RA | 1.4 | 1.4 | 1.5 | n.s. | n.s. | **<0.01** | **<0.01** | **<0.05** | **<0.05** |
| Fibroblast growth factor 23 | FGF23 | 3.0 | 3.0 | 3.2 | n.s. | n.s. | **<0.01** | **<0.01** | **<0.05** | **<0.05** |
| T-cell surface glycoprotein CD8 alpha chain | CD8A | 10.3 | 10.3 | 10.6 | n.s. | n.s. | **<0.01** | **<0.01** | **<0.05** | n.s. |
| Tumor necrosis factor ligand superfamily member 12 | TNFSF12 | 10.0 | 10.0 | 10.1 | n.s. | n.s. | **<0.01** | **<0.01** | n.s. | n.s. |
| Eotaxin | CCL11 | 7.0 | 7.0 | 7.2 | n.s. | n.s. | **<0.05** | **<0.05** | **<0.05** | **<0.05** |
| Leukemia inhibitory factor receptor | LIFR | 4.2 | 4.3 | 4.3 | n.s. | n.s. | **<0.05** | **<0.05** | n.s. | n.s. |
| Neurotrophin-3 | NTF3 | 2.8 | 2.8 | 2.7 | n.s. | n.s. | **<0.05** | **<0.05** | n.s. | n.s. |
| Lymphotoxin-alpha | LTA | 5.3 | 5.4 | 5.2 | n.s. | n.s. | **<0.05** | **<0.05** | n.s. | n.s. |
| C-C motif chemokine 2 | CCL2 | 11.5 | 11.5 | 11.7 | n.s. | n.s. | **<0.05** | n.s. | n.s. | n.s. |
| Fibroblast growth factor 5 | FGF5 | 1.5 | 1.5 | 1.6 | n.s. | n.s. | **<0.05** | **<0.05** | n.s. | n.s. |
| Interleukin-12 subunit beta | IL12B | 6.4 | 6.2 | 6.5 | n.s. | n.s. | **<0.05** | **<0.05** | n.s. | n.s. |
| C-C motif chemokine 25 | CCL25 | 6.4 | 6.4 | 6.5 | n.s. | n.s. | n.s. | n.s. | n.s. | n.s. |
| Urokinase-type plasminogen activator | PLAU | 10.4 | 10.5 | 10.6 | n.s. | n.s. | n.s. | n.s. | n.s. | n.s. |
| Fms-related tyrosine kinase 3 ligand | FLT3LG | 9.8 | 9.9 | 9.9 | n.s. | n.s. | n.s. | n.s. | n.s. | n.s. |
| Fractalkine | CX3CL1 | 6.1 | 6.1 | 6.3 | n.s. | n.s. | n.s. | n.s. | n.s. | n.s. |
| Delta and Notch-like epidermal growth factor-related receptor | DNER | 9.4 | 9.4 | 9.4 | n.s. | n.s. | n.s. | n.s. | n.s. | n.s. |
| Fibroblast growth factor 21 | FGF21 | 5.7 | 5.6 | 5.3 | n.s. | n.s. | n.s. | n.s. | n.s. | n.s. |
| T-cell differentiation antigen CD6 | CD6 | 6.0 | 6.0 | 6.1 | n.s. | n.s. | n.s. | n.s. | n.s. | n.s. |
| Fibroblast growth factor 19 | FGF19 | 8.4 | 8.5 | 8.8 | n.s. | n.s. | n.s. | n.s. | n.s. | n.s. |
| Tumor necrosis factor ligand superfamily member 10 | TNFSF10 | 8.5 | 8.5 | 8.5 | n.s. | n.s. | n.s. | n.s. | n.s. | n.s. |
| Interleukin-17C | IL17C | 1.7 | 1.6 | 1.7 | n.s. | n.s. | n.s. | n.s. | n.s. | n.s. |
| Tumor necrosis factor ligand superfamily member 11 | TNFSF11 | 5.2 | 5.2 | 5.2 | n.s. | n.s. | n.s. | n.s. | n.s. | n.s. |
| Kit ligand | KITLG | 9.8 | 9.7 | 9.8 | n.s. | n.s. | n.s. | n.s. | n.s. | n.s. |
| Interferon gamma | IFNG | 6.6 | 6.6 | 6.7 | n.s. | n.s. | n.s. | n.s. | n.s. | n.s. |
| Interleukin-17A | IL17A | 1.9 | 1.9 | 2.0 | n.s. | n.s. | n.s. | n.s. | n.s. | n.s. |
